# Forensic Post-Mortem Investigation in AAS Abusers: Investigative Diagnostic Protocol. A Systematic Review

**DOI:** 10.3390/diagnostics11081307

**Published:** 2021-07-21

**Authors:** Massimiliano Esposito, Gabriele Licciardello, Federico Privitera, Salvatore Iannuzzi, Aldo Liberto, Francesco Sessa, Monica Salerno

**Affiliations:** 1 Department of Medical, Surgical and Advanced Technologies “G.F. Ingrassia”, Institute of Legal Medicine, University of Catania, 95123 Catania, Italy; massimiliano.esposito91@gmail.com (M.E.); licciardello.gabriele@gmail.com (G.L.); federicoprivitera23@gmail.com (F.P.); driannuzzi@tiscali.it (S.I.); aldo.liberto@gmail.com (A.L.); 2 Department of Clinical and Experimental Medicine, University of Foggia, 71122 Foggia, Italy

**Keywords:** anabolic–androgenic steroids, AASs, adverse effects, cardiac damage, cardiac toxicity, autoptic and histopathological findings

## Abstract

Anabolic–androgenic steroids (AASs) are a group of synthetic molecules derived from testosterone and its precursors. AASs are widely used illicitly by adolescents and athletes, especially by bodybuilders; AASs are among the most used drugs for improving physical performance, as well as for aesthetic purposes. The use of AASs by professional and recreational athletes is increasing worldwide. This review focused on deaths related to AAS abuse and to investigation of the autopsy results and histopathological findings using a rigorous methodology protocol covering: a complete autopsy, histological analysis, and a broad toxicological investigation. Moreover, we aimed to define an investigative diagnostic protocol supporting forensic pathologists during the post-mortem investigation of AAS abusers. This review was conducted using PubMed Central and Google Scholar databases to find articles published between 1 January 1968 and 30 June 2021, using the following key terms: “(anabolic-androgenic steroids) AND (autopsy); (anabolic-androgenic steroids) AND (forensic)”. A total of 939 articles were screened and 926 did not meet the inclusion criteria. In conclusion, 14 articles were included in this systematic review, reporting 137 fatal cases of AAS abuse in total. The histopathologic studies showed myocardial damage characterized by myocyte hypertrophy, focal myocyte damage with myofibrillar loss, interstitial fibrosis, mostly subepicardial, and small vessel disease. Indeed, in AAS-related cases, autopsy plays a pivotal role in the study of AAS adverse effects and organ damage related to their use or abuse. This systematic review aimed to define a specific workflow in death cases related to AASs, suggesting important future insights to better clarify sudden deaths related to AASs, such as the use of miRNAs. The forensic community needs a unified approach in cases of suspected death related to the use of AASs. There are several occasions to apply this workflow, for example in cases of death of bodybuilders and of young people who die in gymnasiums or during sports activities.

## 1. Introduction

**Figure 1:**
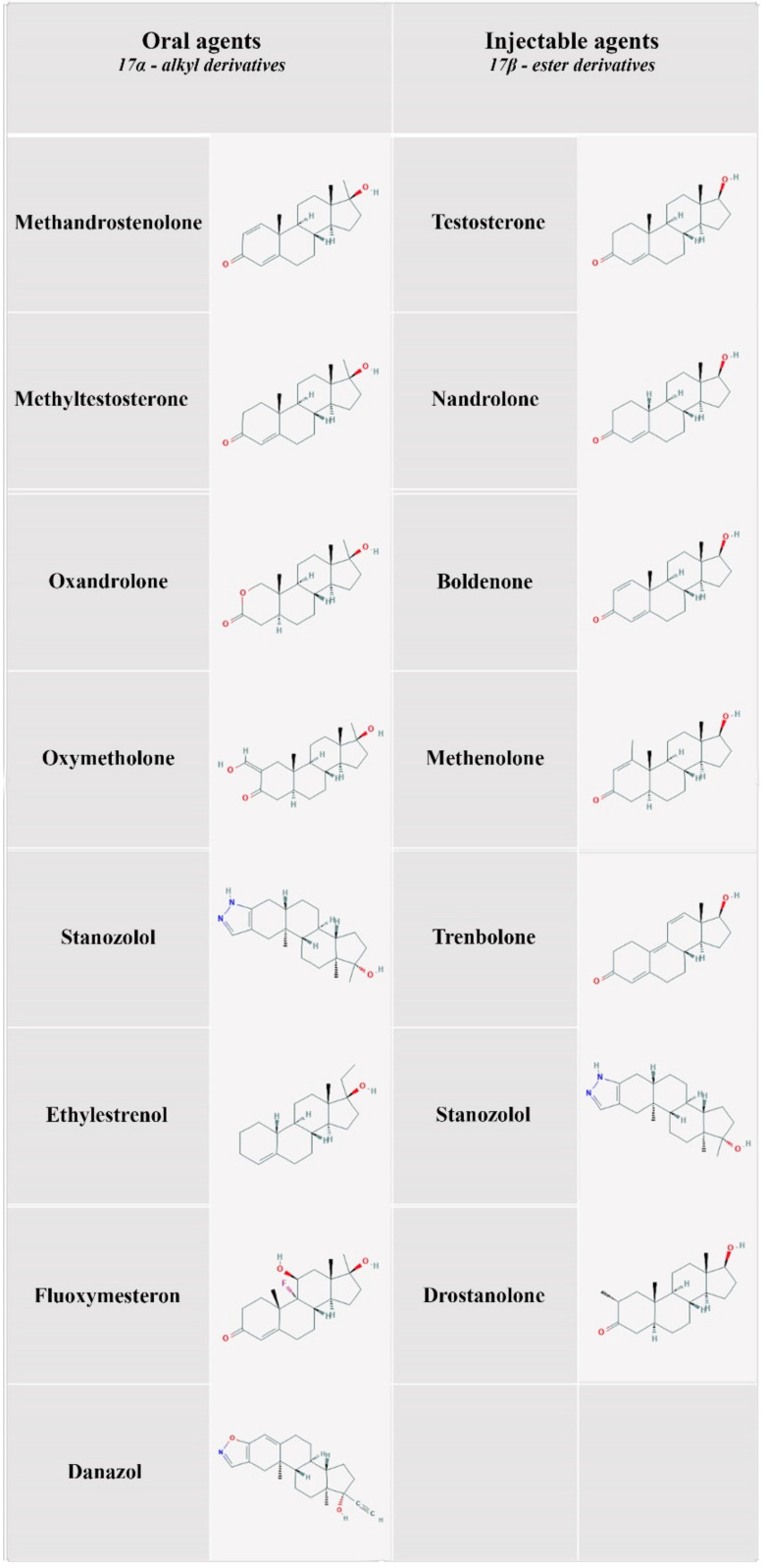
AASs most commonly abused (oral and injectable formulations).

Anabolic–androgenic steroids (AASs) are a group of substances that include testosterone and its synthetic derivatives (Figure 1) [1,2]. AASs were initially developed to minimize the androgenic effects of testosterone and maximize the anabolic effects through the action of growth factor receptors, such as growth hormone (GH) and insulin-like growth factor-1 (IGF-1) [3,4].

Furthermore, AASs and their synthetic derivatives [5] are used at therapeutic dosages in medical practice, in the treatment of hormonal diseases and other pathologies characterized by muscle loss (aging, cancer, and AIDS), hypogonadism, breast cancer, delayed puberty, renal failure, and anemia [6,7,8,9]. Nowadays, the market for doping drugs is huge and constantly increasing. The phenomenon of doping affects not only professional athletes, but also people who perform amateur sports activities (non-athletes), who still have the lowest prevalence of consumption (1%) [10,11]. According to the International Olympic Committee, steroids account for more than 50% of positive doping cases [12]. The Middle East has the highest prevalence of AASs consumption, with 21.7% of world users, followed by South America (4.8%), Europe (3.8%), North America (3.0%), Oceania (2.6%), Africa (2.4%), and Asia (0.2%) [7].

Current legal restrictions are not enough to stop this continuously expanding phenomenon [13].

Furthermore, AAS abuse among non-athletes is increasing especially at fitness centers, for aesthetic purposes [14]. Outside a pharmacological and therapeutic context, AASs are used worldwide among athletes for improving physical performance [15]. Skeletal muscle can be considered as the main target tissue for the anabolic effects of AASs. Self-administration of high doses of AASs is widespread among young athletes to optimize strength and gain muscle mass [16,17]. Nevertheless, exercise is one of the most powerful tools for improving health and has been associated with beneficial changes in most cardiovascular risk factors, including lipids, blood pressure, insulin sensitivity, and weight [18,19].

**Figure 2:**
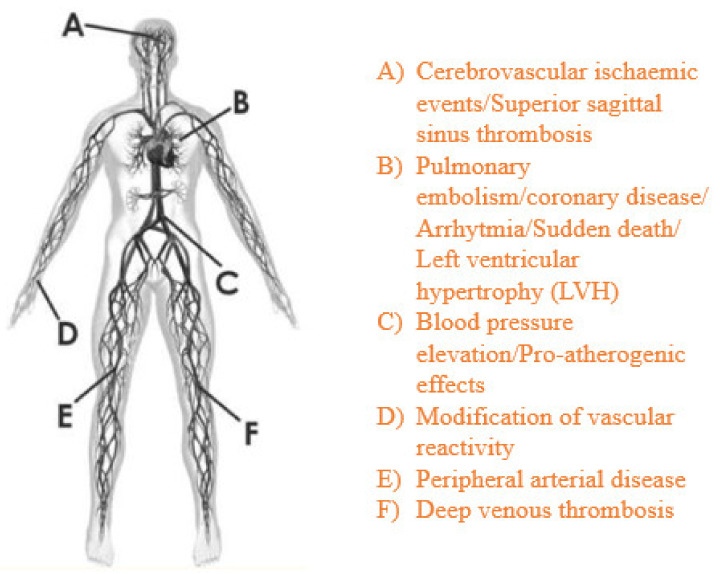
AAS systemic cardiovascular effects.

Several adverse cardiovascular effects of AASs have been reported [20,21,22], such as a decrease in plasma HDL cholesterol levels [23] and sudden death among young athletes, which is a result of cardiovascular diseases, mainly hypertrophic cardiomyopathy [24,25,26,27] (Figure 2). Several adverse effects can involve reproductive, musculoskeletal, endocrine, renal, and hematological systems as well as the liver, and some psychological effects have also been reported [28,29].

Several case reports of sudden death in young athletes indicate a link between chronic AAS abuse and increased risk of arrhythmias and sudden cardiac death [30,31]. Because of the high prevalence of AAS use among athletes, toxicological investigations are fundamental in those cases in subjects suspected of consuming AASs [32,33]. However, the incidence of people dying from AAS abuse is underestimated and few studies have been reported in the literature.

In all cases of sudden death involving a young athlete, the focus of the post-mortem examination should be on the physical phenotype, such as muscular hypertrophy or gynecomastia. In addition, considering that cardiac adverse effects are related to chronic consumption of AASs, and the use of these substances has increased among young adults, cases of sudden death among young adults have increased. Chemical–toxicological analysis is thus a crucial tool to assess the link between sudden cardiac death and AAS abuse [1].

This systematic review focused on forensic autopsies in cases of death related to AAS abuse, analyzing the methods used for each report, in order to provide a methodology for making a faster and more certain death diagnosis. Moreover, this paper aimed to provide the forensic community with a new workflow, suggesting a standard method of investigation to characterize deaths related to the use or abuse of AASs.

## 2. Materials and Methods

A systematic review was conducted according to the Preferred Reporting Items for Systematic Reviews and Meta-Analysis (PRISMA) guidelines [34].

PubMed and Google Scholar were used as search engines to find articles published between 1 January 1968 and 30 June 2021, to evaluate the association between anabolic–androgenic steroid abuse and autopsy findings. The Medical Subject Heading (MeSH) thesaurus was used for the following words: “(anabolic-androgenic steroids) AND (autopsy); (anabolic-androgenic steroids) AND (forensic)”.

### 2.1. Inclusion and Exclusion Criteria

The following exclusion criteria were used: (1) review, (2) articles not in English, (3) abstract, (4) editorial, (5) poster, and (6) communications at conferences. The inclusion criteria were: (1) original article, (2) case report, (3) case series, (4) articles in English, and (5) animal studies.

### 2.2. Quality Assessment and Data Extraction

F.P. and G.L. initially evaluated all the articles, evaluating the title, the abstract, and the whole text. F.P. and G.L. then reanalyzed the articles chosen independently. In cases of conflicting opinions, they were submitted to M.E. and A.L.

### 2.3. Characteristics of Eligible Studies

**Figure 3:**
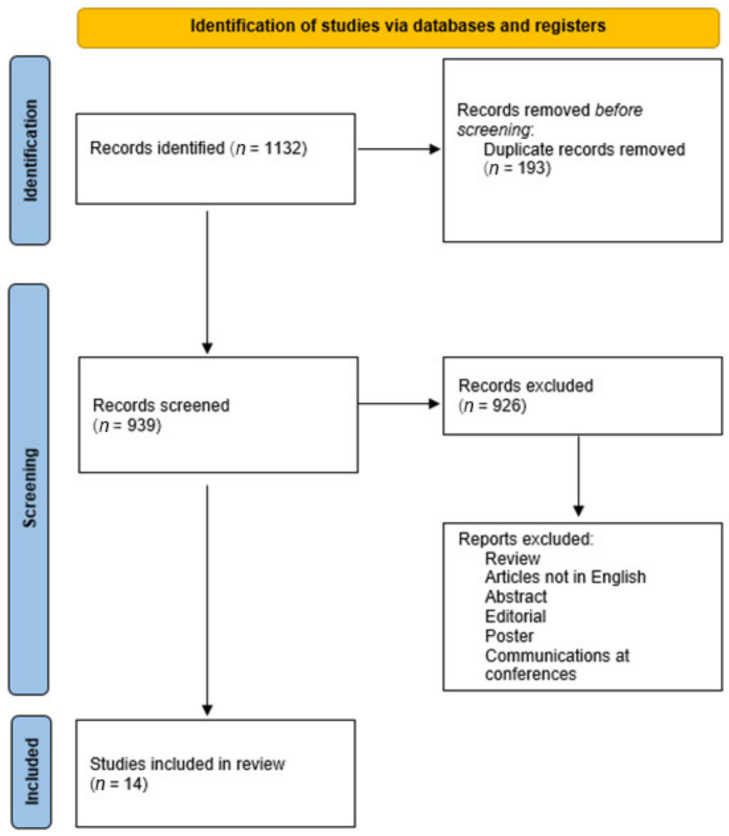
Flow diagram illustrating studies included in and excluded from this systematic review.

A total of 1132 articles were collected. Of these, 193 duplicates were removed. A total of 939 articles were screened and 926 did not meet the inclusion criteria. In conclusion, 14 articles were included in this systematic review (Figure 3). 

## 3. Results

The included studies evaluated in this review were original articles (*n* = 5), case reports (*n* = 6), and case series (*n* = 1). The present study reported a total of 137 fatal cases, most of them were identified in a retrospective study that collected 6 cases of forensic post-mortem examinations performed over a 24-year period in the area of Lyon, France [35], 131 cases came from all the other studies. Routine toxicological analysis was performed using post-mortem samples, collected at autopsy, stored at −18 °C, and used for the identification of testosterone and its synthetic derivatives; furthermore, stanozolol, trenbolone, epitestosterone, and nandrolone were frequently encountered. The most commonly collected samples were urine, hair, and blood, autopsy blood samples were usually taken peripherally from femoral, subclavian vessels. Urine samples taken at autopsy were screened for AAS metabolites, using gas chromatography and mass spectrometry. To perform toxicological investigations in order to ascertain the AASs used, high-performance liquid chromatography (HPLC), gas chromatography–mass spectrometry (GC–MS), headspace gas chromatography with flame-ionization detector (HS–GC–FID) were used. The most used methods were GC–MS (*n* = 8) and HPLC (*n* = 3). It is important to note that the method used for toxicological analysis was not reported in three studies. As summarized in Table 1, the most frequent cause of death was cardiac arrhythmia (ventricular fibrillation) related to left ventricular hypertrophy (LVH) and myocardial fibrosis that create a predisposition to sudden cardiac death. The most significant autopsy or histopathological findings on AAS abusers were concentric cardiac hypertrophy associated with coronary thrombosis and LVH, fibroblasts, cardiac proliferation, and myocytolysis. In several studies, different cardiac patterns were described, with the presence of single necrosis with homogeneous eosinophilia, myofibrillar degeneration, contraction bands, and cytoplasm. Another reported characteristic finding was the presence of interstitial edema. Occasionally, hypercontraction of cardiomyocytes with heavily thickened Z-lines was reported on hematoxylin and eosin staining. Other findings were short sarcomeres with pathological cross bands and irregular eosinophils consisting of segments with hypercontracted or coagulated sarcomeres. In other cases, the histological investigation of the heart showed a total interruption of myofibrils with a granular appearance of the whole cell without well-defined pathological bands. Immunohistochemical analysis was conducted, in most of the studies, through antibodies against troponin T, fibronectin, and myoglobin, confirming fibrosis and heart cells inflammation. In only one case was there an overstimulation of the sympathetic nervous system followed by a transient functional and structural destabilization of the sympathetic terminal axons. Subsequently, a ventricular fibrillation was reported. In the same case series, the autopsy findings supported by histopathological investigation highlighted hepatic injury, including cholestasis and peliosis hepatis [36].

## 4. Discussion

AASs are used worldwide among athletes for improving physical performance [15]. The prolonged misuse and abuse of AASs is linked to various pathological alterations, related to dose, frequency, and patterns of use. Several adverse effects can involve cardiovascular, reproductive, musculoskeletal, endocrine, renal, and hematological systems as well as the liver, and some psychological effects have also been reported [28]. Some of these adverse effects may be fatal, especially the ones regarding the cardiovascular system, such as sudden cardiac death and coronary artery disease [28,29]. It was described that cardiovascular disease was widespread in AAS-related deaths [43], as were asymmetrical left ventricular hypertrophy, coronary atherosclerosis causing significant luminal narrowing, pulmonary thromboembolism, coronary and endocavitary thrombi, inflammatory infiltrates, and cardiomegaly (diagnosed by comparing the weight of the heart with the body weight and BMI of the patient) [47,48,49].

Furthermore, histopathologic studies showed myocardial damage characterized by myocyte hypertrophy, focal myocyte damage with myofibrillar loss, interstitial fibrosis (mostly subepicardial), and small vessel disease [21].

In toxicology samples collected during autopsies, testosterone and its synthetic derivatives were identified. In addition, abnormalities in steroid profiles were examined for evidence of exogenous testosterone use, with a testosterone/epitestosterone ratio of >6:4 [44,45,46], a common feature of AAS users. (A ratio of >6:1 in males and a ratio of >4:1 in females are interpreted as highly indicative of exogenous testosterone administration.) Collecting different kinds of samples is essential for a correct post-mortem evaluation; for example, keratin matrices of a 1 cm long hair sample can detect substances over a period from 4 to 30 days prior to the autopsy; otherwise, AASs are not always detectable in femoral blood, stomach content, and urine.

Liquid biological samples (blood and urine) should undergo a screening using chromatography–mass spectrometry (GC–MS). Urine samples should be first buffered to pH 7.0 and hydrolyzed using β-glucuronidase from *Escherichia coli*. The pH is then adjusted to a value of 9.5 and the analytes are extracted through tertbutylmethylether (MTBE). If the results are negative, they can either be subjected to GC–MSor to liquid chromatography–tandem mass spectrometry (LC–MS–MS) again to confirm the onset of AAS [50]. For blood analyzes, liquid chromatography–mass spectrometry (LC–MS) is used to obtain the precipitation of proteins [37,51].

Another forensic toxicological approach to AAS measurement involves the extraction of their phase II metabolites. This process could increase the sensitivity of the technique. A phase II metabolite of methandienone was recently analyzed and the authors stated that it could be used as a long-term marker of this substance (up to 26 days after single oral administration) [52,53]. In addition to liquid biological samples, the toxicological analysis of AAS can also be performed on hair. The extracted hair should be decontaminated in two dichloromethane baths (5 mL, 2 min). The organic phase is obtained after centrifugation and should be collected, evaporated, and diluted with phosphate buffer pH 7.0 (part a). The hair remaining on the bottom of the tube is collected and then hydrolyzed with 1 mL of 1 M NaOH (part b). In both parts (a and b), the liquid–liquid extraction is performed with 2 mL of ethyl acetate. After further processing, androstenedione, testosterone, boldenone, tetrahydrogestrinone, methandienone, methenolone, methyltestosterone, nandrolone, oxandrolone, chlorodehydromethyltestosterone, stanozolol, and trenbolone should all be tested by LC–MS–MS. Esters (testosterone, nandrolone, and drostanolone) should be tested by GC–MS–MS [54].

Consistent with these studies [51,52,53], the results presented in this review show that the most frequent method of analysis was GC–MS (followed by HPLC), highlighting how much reliable this method is for investigating deaths related to AAS abuse.

MicroRNAs (miRNAs) are a group of 20–22 nucleotides-long non-coding RNAs that regulate gene expression by inhibiting translation of their target messenger RNAs (mRNAs) [55,56,57]. The use of miRNAs has important advantages over the use of other nucleic acids. First, mature miRNAs are much more stable than other nucleic acids; this is of crucial importance in the forensic field because it allows better preservation of unaltered evidence. This would also apply to formalin-desiccated and paraffin-embedded tissues, where nucleic acid fractionation occurs. Furthermore, miRNA profiling has greater discriminatory potential than other nucleic acids. Finally, specific body fluid miRNA-detection assays have been created for blood (including menstrual), urine, semen, vaginal secretions [58].

It was demonstrated that AASs increase the risk of premature death, especially in subjects with other pathologies and/or psychiatric diseases [43]. Tseng et al. [59] conducted a study about out-of-hospital cardiac arrest (OHCA) and sudden cardiac death (SCD). To verify if the out-of-hospital deaths were due to cardiac causes, autopsy with histological and toxicological investigations were performed, as it was recently suggested to ascertain the exact cause of death in suspected SCD [60]. A multidisciplinary commission judged the final cause. An important finding was that the second cause of OHCA after coronary artery disease was an occult substance overdose. These data were discovered through toxicological investigation. This is consistent with one of the conclusions of this review—a toxicological investigation is crucial in deaths from suspected AAS abuse. A survey conducted in 21 gymnasiums in Britain reported that 8% of respondents declared having taken AASs in their life. Another study in the UK showed that 70% of the clientele in a health and fitness community were AAS users [21].

The combination of physical activity and prolonged, chronic, or previous misuse of AASs leads to a predisposition to different patterns of myocardial injury and SCD [28], which is generally defined as sudden unexpected death or arrest from a presumed cardiac cause, which occurs within one hour of symptom onset if witnessed, otherwise within 24 h, in a person without any prior condition that would appear fatal [61,62,63].

Melchert and Welder [64] categorized the effects of AASs on the cardiovascular system into four groups: vasospastic, atherogenic, thrombotic, and direct myocardial injuries. AASs can induce adverse cardiovascular effects, such as left ventricular hypertrophy (LVH), hypertension, impaired diastolic filling, arrhythmia, erythrocytosis, thrombosis, and altered lipoprotein profiles. It is possible to evaluate apparently healthy subjects who chronically use supraphysiological doses of AASs, using SAECG (signal-averaged electrocardiography), providing diagnostic and prognostic information on the risk of arrhythmias and SCD [65,66,67,68].

Nandrolone is a testosterone derivative, known as one of the most commonly used androgens and anabolic steroids to improve athletes’ physical performance, exhibiting strong anabolic effects and weak androgenic effects [69,70,71,72].

Testosterone is the major regulator of the hypothalamic–pituitary–testicular axis; it is not surprising that exogenous testosterone and AASs exert a suppressive effect on the hypothalamic–pituitary system. The resulting suppression of luteinizing hormone (LH) and follicle-stimulating hormone (FSH) leads to a decrease in intratesticular testosterone and secreted testosterone, as well as to a decrease in spermatogenesis and sperm production. Varying doses, preparations, and combinations of AASs make it difficult to draw general conclusions from individual observations, but it is clear that the recovery of sperm counts correlates positively with the time since the last intake of AASs, as do sperm morphology and motility [73,74]. Therefore, it may be useful to collect gonad samples during autopsy for further histological examinations.

Animal studies are important as they confirm that AASs act directly on heart cells by changing conformation and causing direct damage that can lead to sudden cardiac death. Indeed, studies on isolated hearts from rats treated chronically with nandrolone decanoate (ND) have also shown a rise in myocardial susceptibility to ischemia–reperfusion injuries [75,76,77]. Rocha et al. [78] studied the effects on cardiac function in rats undergoing swimming training and those not undergoing it. They found that swimming training combined with high doses of nandrolone (5 mg/kg per injection, equal to 10 mg/kg per week) aggravates cardiac hypertrophy with interstitial fibrosis. A recent study showed that chronic nandrolone treatment with or without severe training causes a significant increase in beta-myosin heavy chain (β-MHC) gene expression, calcium/calmodulin-dependent protein kinase II (CaMKII), and monoamine oxidase (MAO) activities in the heart tissue of male Wistar rats [79].

It is difficult to distinguish the etiology of these changes from histological findings alone, and it becomes essential to evaluate the subject’s clinical history and physical characteristics in all cases of sudden cardiac death in which AAS abuse is suspected [80,81,82]. The physical phenotype of a male who abuses AASs includes characteristics such as muscle hypertrophy, prominent striae above the pectoralis or biceps muscle, breast development (gynecomastia), testicular atrophy, and severe acne. In women, signs of AAS use also include hirsutism, deepening of the voice, and masculinization of secondary sexual characteristics [83,84].

The focus of our systematic review concerns the autopsy findings related to AAS abuse, but there is considerable evidence in the international literature of non-fatal systemic adverse effects, which can guide the medico-legal study of any single case. For example, prolonged administration of AASs is associated with aggressive behavior, related to an altered molecular expression of ERα or ERβ receptors in regions of the brain responsible for the control of aggression [85]; in another report, AAS abuse induces testicular damage by triggering oxidative stress via inflammatory cytokines, matrix metalloproteinases, cell adhesion molecules, apoptotic markers, and DNA damage [86]; another study demonstrated that the chronic use of AASs, combined with a high-protein diet, can create severe renal damage, such as focal segmental glomerulosclerosis (FSGS), nephroangiosclerosis, chronic interstitial nephritis, and acute interstitial nephritis [87].

Taking the results of this systematic review into consideration, it is possible to be consistent with the academic literature on AAS users. In the majority of cases, common autopsy and histopathological findings were concentric cardiac hypertrophy with enlargement of the ventricular walls as well as a small vascular lumen, left ventricular hypertrophy (LVH), coronary thrombosis, and proliferation of fibroblasts consistent with early fibrosis [35,37,38,39,40,41,42]. In only two cases, overstimulation of the sympathetic nervous system was the trigger that led to ventricular fibrillation [36].

**Figure 4:**
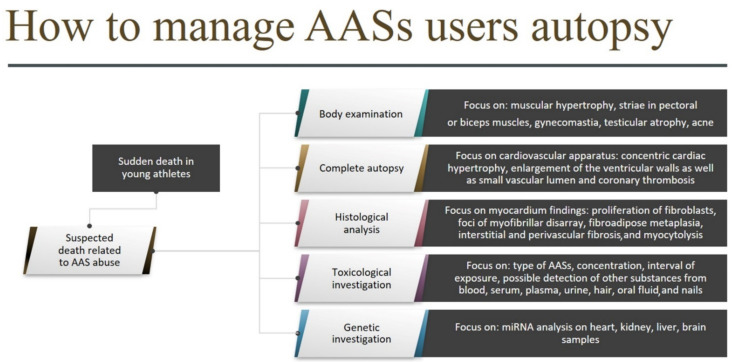
Investigative protocol for cases of suspected death related to AAS abuse.

Based on this systematic review, it is possible to hypothesize a workflow in cases of death related to AAS abuse (Figure 4). The relationship between AAS abuse, vigorous exercise training, and cardiac death can be evaluated only by the application of an investigative protocol, which must include a rigorous methodology covering:

- A complete autopsy with special regard to AAS target organs and apparatus (the cardiovascular system above all);
- Histological analysis of AAS target organs, with a focus on concentric cardiac hypertrophy, coronary thrombosis, left ventricle hypertrophy (LVH), fibroblasts cardiac proliferation, and myocytolysis;
- A broad toxicological investigation, preceded by a careful evaluation of clinical-anamnestic data, to confirm AAS consumption (including the type of AAS, concentration, and interval of exposure) and possible detection of other substances that could have contributed to the fatal outcome. For this purpose, different matrices can be used; urine is the most common because it provides a prolonged detection window, but several other matrices, such as blood, serum, plasma, hair, oral fluid, and nails can also be used; in addition, gonad samples could be useful to detect early adverse effects, such as hypogonadism or azoospermia [74,88].

When an autopsy is performed in a sudden death case involving a young athlete, the focus of the examination should be on the physical phenotype, such as muscular hypertrophy, striae in pectoral or biceps muscles, gynecomastia, testicular atrophy, and acne. To suggest AAS abuse, a complete and detailed autopsy of all the cardiovascular apparatus, with a focus on the heart, is mandatory. The chemical–toxicological analysis is a crucial tool to assess the link between sudden cardiac death and AAS abuse [1]. Autopsy plays a pivotal role in the study of AAS adverse effects and organ damage related to their use/abuse. Moreover, autopsy studies may provide useful information regarding the pathophysiology of the effects of long-term administration of AASs; therefore, autopsy practice should be implemented in suspected AAS-related deaths [89,90]. The forensic community needs a unified approach in cases of suspected death related to the use of AASs. There are several occasions to apply this workflow, for example in cases of bodybuilders and young people who died in gymnasiums or during a sport activity.

## 5. Conclusions

The comparison of the cases reviewed here, in agreement with previous studies already present in the literature, support the hypothesis that the combined effects of strong workout, prolonged, chronic or previous abuse of AASs in different forms and combinations create a predisposition to develop pathological patterns of myocardial injuries and, consequently, sudden cardiac death [90]. The problem of AAS use and abuse is still relevant, even though their use is illegal and despite the large and increasing number of sudden cardiac deaths among young subjects. There is still no complete understanding of the real dangers of using these substances.

Clinicians should pay more attention to early signs indicative of AAS use and consider those physical and epidemiological characteristics that can lead to the suspicion of AAS abuse to implement primary prevention measures. Therefore, the purpose of this systematic review is both to guide forensic pathologists through a more accurate methodologic approach based on autoptic and histological examinations and to emphasize the necessity to reinforce the “global warning” (already expressed in previous reports) against the use and abuse of these substances among professional and non-professional athletes [36]. An interesting challenge would be to further investigate these findings, to be able to use these biomarkers both to facilitate the post-mortem diagnosis of sudden deaths related to AAS abuse and as a screening method in living subjects to prevent fatal consequences.

This systematic review revealed that to date, there are not many cases of the use of new genetic markers (miRNA) to better define specific damage caused by AASs that could lead to sudden death. These new genetic techniques not only improve the accuracy of forensic determination of AASs as the cause of death but could help the scientific community to understand some of the aspects related to AASs abuse that are still unknown. A recent review confirmed the pivotal role of genetic investigation in the case of SCD [60]. Moreover, several recent studies reported the importance of research in the field of new molecular biomarkers such as miRNAs [23,91]. Thanks to these advances, it could be possible to define new diagnostic tools in order to ascertain asymptomatic heart diseases. To date, the implementation of new technologies, such as next-generation sequencing (NGS), may improve the results increasing the number of application fields in the management of SCD.

## Figures and Tables

**Table 1:** Summary of the details of the systematic review.

Reference	Study Design	Number of Cases	Autopsy/Histopathological Findings	Immunohistochemical Findings	AASs/Toxicological Analysis Samples	Toxicological Method	Cause of Death
Lehmann S. [37]	Case report	1 young adult	Hypertrophy of the heart with enlargement of the ventricular walls as well as a small vascular lumen (3 mm diameter) of the right coronary artery	Immunohistochemistry staining was conducted with antibodies against primary antibodies against troponin T that showed inflammation, fibrosis or necrosis	- Testosterone, oxymetholone, stanozolol, trenbolone - Blood, urine, cerebrospinal fluid, and stomach content	HPLC	Pathological changes of the heart (left ventricular hypertrophy) and atherosclerosis of the coronaries
Bertozzi G. [6]	Case report	1 young adult	Left thigh necrotizing myofasciitis	Immunohistochemistry with anti-myoglobin antibodies showed myofibrillar rexis on heart samples	- Testosterone, propionate, clenbuterol, stanozolol, trenbolone, oxandrolone, tamoxifen - Hair and blood	GC–MS	AASs adversely influenced the immune response, affecting leucocyte growth or activity, and antibody and cytokine production
Lichtenfeld J. [38]	Case report	1 young adult	Left ventricular myocardium findings: foci of myofibrillar disarray, proliferation of fibroblasts consistent with early fibrosis, and enlarged myofibers with heterogeneity of nuclear size including box car nuclei	Not available	- Standard urine toxicology tests were negative	Not available	Cardiac arrest attributed to hypertrophic cardiomyopathy from anabolic steroid use, with documented ventricular fibrillation as the initiating arrhythmia
Fineschi V. [36]	Case series	2 young adults	Hepatic injury, including cholestasis, peliosis hepatis, hyperplasia, ventricular fibrillation	Not available	- Testosterone, nandrolone, norandrosterone, etiocholanolone, noretiocholanolone, norepiandrosterone, stanozolol, hydroxy-stanozolol, epitestosterone - Urine	GC–MS	Overstimulation of the sympathetic system followed by a transient functional and structural destabilization of the sympathetic axon terminals
Fanton L. [35]	Original article (retrospective study)	6 of 2250 young adults	Coronary thrombosis associated with left ventricle hypertrophy, toxic, adrenergic myocarditis, dilated cardiomyopathy associated with a recent coronary thrombosis	Immunohistochemical staining was conducted with hematoxylin–phloxin–saffron (HPS) antibodies, which showed interstitial inflammatory cells, interstitial reticular fibrosis, concentric stenosing and intimal sclerosis of the heart	- Norethandrolon - Blood	GC–MS	Various cardiac lesions: misshapen cell nuclei, myolysis, fibrosis and interstitial lesions
Lusetti M. [39]	Original article (retrospective study)	6 of 98 young adults	Pathological changes consisted of various degrees of interstitial and perivascular fibrosis as well as fibroadipose metaplasia and perineural fibrosis within the myocardium of the left ventricle	Immunohistochemistry, antibodies against fibronectin and c5b9 showed a myocardium fibrosis	- Testosterone, nandrolone, epitestosterone - Cardiac blood, vitreous humor, urine, cerebrospinal and pericardial fluids as well as gastric contents, hair and samples of certain tissues (liver, brain, and skeletal muscle)	GC–MS	Left ventricular hypertrophy and myocardial fibrosis can create a predisposition to sudden cardiac death
Montisci M. [40]	Case report	4 young adults	Concentric cardiac hypertrophy with focal fibrosis (one case), dilated cardiomyopathy with patchy myocyte death (two cases) and eosinophilic myocarditis (one case). The most typical cardiac abnormality in AAS abusers is left ventricular hypertrophy, associated with fibrosis and myocytolysis	Immunohistochemical analysis through primary antibody against Troponin T showed myocytolysis in the sub-endocardial trabeculae, hypertrophic myocytes with dysmetric and dysmorphic nuclei	- Testosterone, stanazolol, nortestosterone, epitestosterone - Hair and urine	GC–MS	Three cases of sudden cardiac death (SCD) and one of death due to congestive heart failure of a previously healthy athlete
Inoue H. [41]	Case report	1 young adult	Concentric cardiac hypertrophy was macroscopically observed. In the left and right coronary arteries, atherosclerosis was generally observed within the endothelium	Immunohistochemical analysis through primary antibody against Troponin T showed myocytolysis in the sub-endocardial trabeculae, hyper-trophic myocytes with dysmetric and dysmorphic nuclei	- Testosterone - Blood	Not available	Ischemic heart disease due to coronary stenosis
Far M. [42]	Original article (retrospective study)	87 of 260 adults (1989 to 2009)	A significantly greater cardiac mass among deceased users of AASs compared to individuals with no suspected use of AASs. An elevated risk of developing concentric LVH among AAS users	Not available	- Testosterone, propionate, clenbuterol, stanozolol, trenbolone, oxandrolone, tamoxifen - Urine	GC–MS	Cardiac hypertrophy with a direct cardiotropic effect
Darke S. [43]	Original article (retrospective study)	24 adults (1995–2012)	In 23 of 24 cases, substances other than steroids were detected, most commonly psychostimulants (66.7%); in nearly half, testicular atrophy was noted, as was testicular fibrosis and arrested spermatogenesis; left ventricular hypertrophy was noted in 30.4%, and moderate to severe narrowing of the coronary arteries in 26.1%	Not available	- Testosterone, epitestosterone - Blood, urine	HPLC	Particularly notable extensive cardiovascular disease
Thiblin I. [44]	Case report	1 young adult	Both the foci of replacement fibrosis and the perivascular inflammatory changes were rather moderate, and probably not severe enough to cause arrhythmia by themselves, both fibrosis and myocardial inflammation are known risk factors for arrhythmia	Not available	- Testosterone, OH-stanozolol, - 16b-OH-stanozolol, boldenon - Blood, urine	GC–MS	Sudden cardiac arrhythmia possibly related to a combination of AASs and ephedrine
Hernández-Guerra, A. I. [1]	Case report and literature review	1 young adult	Cardiomegaly (420 g) with a ventricular thickness that was within the upper normal ranges (left ventricular free wall 15 mm, ventricular septum 15 mm, right ventricular free wall 5 mm); acute myocardial infarction at the anterior third of the septum and the left ventricle (LV) anterior wall, subacute myocardial infarction at apical septum and apical posterior LV wall	Immunohistochemical analysis with primary antibodies against troponin T showed small intramyocardial vessels disease with media hypertrophy	- Stanozolol, testosterone, tamoxifen, mesterolone, nandrolone - Blood, humor	HS–GC–FID	Myocardial infarction with severe coronary atherosclerosis and acute occlusive thrombosis affecting left main trunk and left anterior descending coronary artery (LAD) (single vessel disease)
Dufayet L. [45]	Case report	1 young adult	Yellow discoloration of the skin, nonspecific signs of asphyxia (cyanosis, pulmonary edema and congestion); Heart: occasional foci of vascular congestion in the connective tissue surrounding coronary arteries; Lungs: edematous and congestive, with some areas of alveolar hemorrhage; Mild congestion was alsoobserved in the centrilobular region of the liver as well as in both kidneys.	Immunohistochemical analysis through primary antibody against Troponin T revealed occasional foci of vascular congestion in the connective tissue surrounding coronary arteries	- 2,4-dinitrophenol (DNP) clenbuterol - Blood - Urine - Gastric content	GC– MSHPLC	Toxicological analysis showed high levels of clenbuterol and DNP, confirming an intoxication
Dickerman R. D. [46]	Case report	1 young adult	The heart weighed 250 g with signs of concentric hypertrophy of the left ventricle, atherosclerosis of the vessels	Not available	- Testosterone, - nandrolone	Not available	Cardiac hypertrophy with a direct cardiotropic effect

## Data Availability

Data sharing not applicable, no new data were created or analyzed in this study.
